# Feasibility of abbreviated cycles of immunochemotherapy for completely resected limited-stage CD20+ diffuse large B-cell lymphoma (CISL 12-09)

**DOI:** 10.18632/oncotarget.14531

**Published:** 2017-01-05

**Authors:** Dok Hyun Yoon, Byeong Seok Sohn, Sung Yong Oh, Won-Sik Lee, Sang Min Lee, Deok-Hwan Yang, Jooryung Huh, Cheolwon Suh

**Affiliations:** ^1^ Department of Oncology, Asan Medical Center, University of Ulsan College of Medicine, Seoul, Korea; ^2^ Department of Internal Medicine, Sanggye Paik Hospital, Inje University College of Medicine, Seoul, Korea; ^3^ Department of Internal Medicine, Dong-A University Hospital, Busan, Korea; ^4^ Department of Hemato-Oncology, Internal Medicine, Busan Paik Hospital, Inje University College of Medicine, Busan, Korea; ^5^ Department of Hematology-Oncology, Chonnam National University Hwasun Hospital, Hwasun, Korea; ^6^ Department of Pathology, Asan Medical Center, University of Ulsan College of Medicine, Seoul, Korea

**Keywords:** diffuse large B-cell lymphoma, limited stage, complete resection, abbreviated cycles and R-CHOP chemotherapy

## Abstract

**Background:**

The appropriate number of chemotherapy cycles for limited stage diffuse large B-cell lymphoma (DLBCL) patients without gross residual lesions after complete resection, has not been specifically questioned. We performed a multicenter, single-arm, phase 2 study to investigate the feasibility of 3 cycles of abbreviated R-CHOP chemotherapy in low-risk patients with completely resected localized CD20+ DLBCL.

**Results:**

Between December 2010 and May 2013, we recruited 23 patients. One was excluded due to ineligibility, and hence, 22 were included in the final analysis. The primary sites comprised the intestine (*n* = 15), cervical lymph nodes (*n* = 4), stomach (*n* = 1), tonsil (*n* = 1), and spleen (*n* = 1). All patients successfully completed the 3 cycles of planned R-CHOP chemotherapy. Over a median follow-up of 39.5 months (95% confidence interval, 29.9—47.1 months), both the estimated 2-year disease-free survival and overall survival rates was 95% confidence interval, 85.9–104.1%. Only one patient with an international prognostic index of 2 experienced relapse and died. The most common grade 3 or 4 toxicity condition included neutropenia (*n* = 8, 36.4%). Three patients experienced grade 3 febrile neutropenia, but no grade 3 or 4 non-hematologic toxicity was observed.

**Materials and Methods:**

DLBCL patients without residual lesions after resection were enrolled and R-CHOP chemotherapy was repeated at 3-week-intervals over 3 cycles. The primary endpoint was 2-year disease-free survival.

**Conclusions:**

Three cycles of abbreviated R-CHOP immunochemotherapy is feasible for completely resected low risk localized DLBCL.

## INTRODUCTION

A full course of R-CHOP chemotherapy or an abbreviated course of R-CHOP chemotherapy followed by radiotherapy, is recommended as the standard of care for limited stage (LS) diffuse large B-cell lymphoma (DLBCL). Using this combined treatment modality, the 2-year progression-free survival (PFS) rate has been improved to up to 93% in certain LS DLBCL patients [1]. Moreover, the British Columbia Cancer Agency group showed that 3 cycles of abbreviated R-CHOP chemotherapy, followed by an additional cycle of chemotherapy, while omitting radiotherapy, can achieve excellent outcomes in elderly patients with LS DLBCL, who were found to achieve negative interim ^18^F-fluorodeoxyglycose positron emission tomography (FDG-PET) [2].

In some cases, lymphomatous lesions are completely resected during the diagnostic surgical procedure. In addition, surgical resection of the lesions is often performed in cases of gastrointestinal lymphomas with LS disease due to the presence of obstructive lesions, uncontrolled bleeding, or perforation risk. In these completely resected patients, the subsequent chemotherapy aims to not treat the macroscopic lesions but only eradicate any microscopic disease if present. However, in these patients without gross residual lesions, the adequate number of chemotherapy cycles remains unexplored. Hence, full cycles of chemotherapy are usually administered by following the conventional treatment scheme in advanced stage disease. Thus, we aimed to investigate the feasibility of a less intensive therapeutic approach—a 3-cycle abbreviated course of R-CHOP chemotherapy—in low-risk patients with completely resected stage I or II CD20+ DLBCL.

## RESULTS

### Patient characteristics

Between December 2010 and May 2013, we enrolled 23 patients. Of these, we included 22 patients in the analysis excluding 1 patient with ineligibility. The baseline characteristics are summarized in Table 1. The preoperative lactate dehydrogenase (LDH) level was available for 11 patients, as the study procedures were initiated after resection; It was elevated in 2 patients, in whom the level was still above the reference range after surgical resection. Thus, preoperative international prognostic index (IPI) scores could be calculated in those 11 patients, and were found to be 0 in 8, 1 in 2, and 2 in 1 patient. Among the 11 patients for whom preoperative LDH levels were not available, 8 were elderly without other risk factors for IPI and 10 had at least 1 risk factor of stage-modified IPI [3], either age (*n* = 8) or stage 2 disease (*n* = 2). The postoperative LDH levels of these patients were within the reference range. One patient with unknown IPI had CD5-positive DLBCL. According to the cell of origin (COO) based on Han's criteria [4], 10 patients were classified as having germinal-center B-cell like (GCB)-DLBCL and the other 12 were classified as having non-GCB DLBCL.

**Table 1:** Patient characteristics

Clinical characteristics	*N* = 22
Time to chemotherapy after resection, days (median, range)	24 (9–49)
Age, years (median, range)	57 (29–77)
≤ 60	12 (54.5%)
> 60	10 (45.5%)
Sex	
Male	18 (81.8%)
Female	4 (18.2%)
ECOG performance status,	
0–1	22 (100%)
Ann Arbor stage	
1/1E	14 (63.6%)
2E*	8 (36.4%)
Lactate dehydrogenase, preoperative	
Within normal limits	9 (40.9%)
Increased	2 (9.1%)
NA	11 (50.0%)
IPI	
0	8 (36.4%)
1	2 (9.1%)
2	1 (4.5%)
NA	11 (50.0%)
Stage-modified-IPI	
0	3 (13.6%)
1	6 (27.3%)
2	2 (9.1%)
NA	11 (50.0%)
Involved sites and surgical procedures	
Intestine	15 (68.2%)
Appendectomy	1
Hemicolectomy	5
Ileocecectomy	7
Small bowel resection	2
Stomach, distal gastrectomy	1 (4.5%)
Tonsil, tonsillectomy	1 (4.5%)
Spleen, splenectomy	1 (4.5%)
Cervical LNs, LN excision	4 (18.2%)
Cell of origin according to Han's criteria	
GCB	10 (45.5%)
Non-GCB	12 (54.5%)

Abbreviation: ECOG, Eastern Cooperative Oncology Group; GCB, germinal-center B-cell like; IPI, International prognostic index; LN, lymph node; NA, not available

*All the patients with stage 2 had intestinal lymphoma with regional lymph node involvement.

### Treatment and survival outcomes

All of the patients included in the analysis completed the planned 3 cycles of R-CHOP chemotherapy. The median time from resection to chemotherapy was 24 days (range, 9–49 days); 2 patients started chemotherapy on day 45 and 49, after a 6-week interval, due to delayed recovery from surgery. Three elderly patients received dose-attenuated R-CHOP from the first cycle. Two of these patients required another dose reduction by 25% for cyclophosphamide and doxorubicin from the second cycle due to the development of grade 4 neutropenia. Another 4 patients required dose reduction for cyclophosphamide and/or doxorubicin during treatment due to the development of grade 4 neutropenia (*n* = 2) or grade 3 febrile neutropenia (*n* = 2). None of the patients required dose modification for rituximab, vincristine, or steroids. Thus, the mean cumulative dose of each agent is as follows: 1,125 mg/m^2^ for rituximab, 2,062.5 ± 311.6 mg/m^2^ for cyclophosphamide, 139.3 ± 20.2 mg/m^2^ for doxorubicin, 6 mg for vincristine, and 300 mg for prednisolone. The cyclophosphamide and doxorubicin doses correspond to 45.8% and 46.4% of those used for 6 cycles of R-CHOP. All patients were confirmed as having no evidence of disease via PET-CT after 3 cycles of R-CHOP chemotherapy, except for 2 patients who refused response evaluation. However, these 2 patients were confirmed to be alive and well at the final survival data update (July 2016).

**Figure 1:**
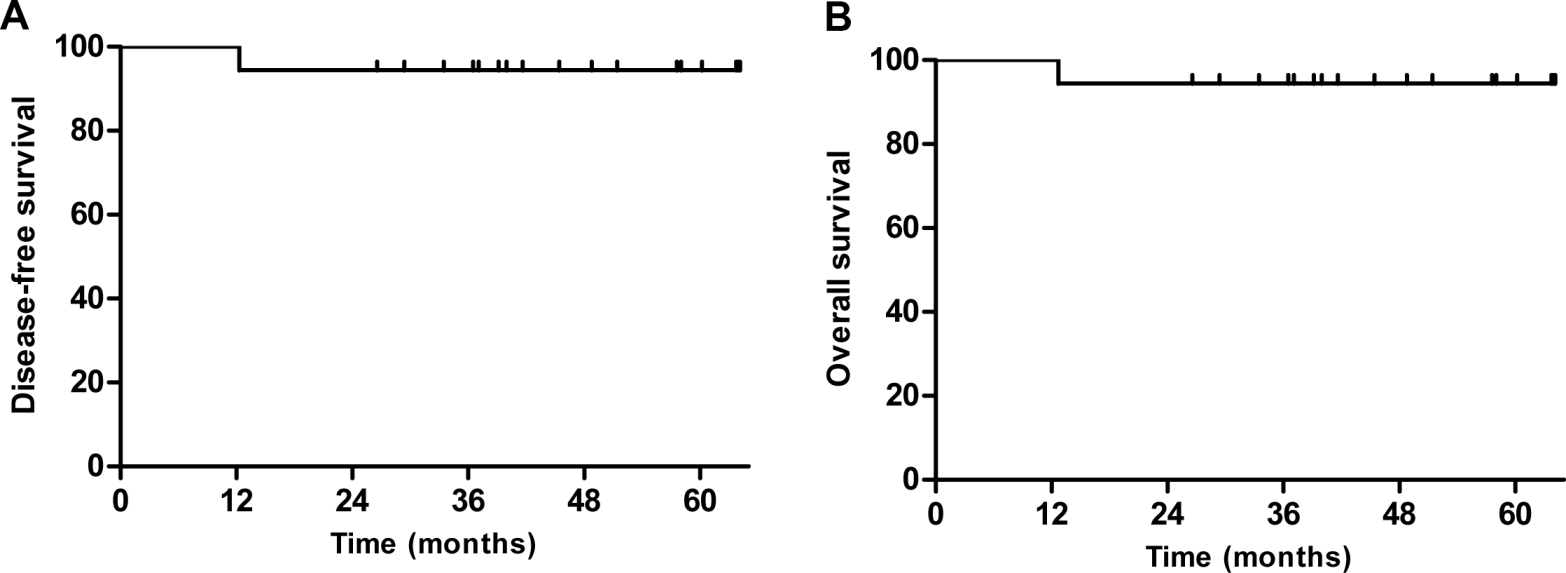
Kaplan-Meier curves of (A) disease-free survival (DFS) and (B) overall survival (OS) The estimated 2-year DFS and OS rates were 95.0% (95% confidence interval, 85.89–104.11%)

Over a median follow-up duration of 39.5 months (95% confidence interval [CI], 29.9–47.1 months), only 1 patient showed disease progression. This patient was a 68-year-old man with non-GCB type disease; he was the only patient with a preoperative IPI of 2 (aged > 60 years and elevated LDH levels). He had 2 splenic nodules and underwent splenectomy, followed by 3 cycles of R-CHOP chemotherapy. Systemic disease progression was confirmed at 12.3 months, and he died due to rapid progressive disease after 2 weeks despite salvage R-ESHAP chemotherapy. The estimated 2-year DFS and OS rates were 95.0% (95% CI, 85.9–104.1%; Figure 1). All of the 16 patients with gastrointestinal tract involvement survived without disease progression (Supplementary Materials, Supplementary Table 1, and Supplementary Figure 1).

### Toxicity

The toxicities are summarized in Table 2. Neutropenia was the most common grade 3 or 4 hematologic toxicity (36.4%). Three patients experienced grade 3 febrile neutropenia, all of which developed after the first cycle of R-CHOP chemotherapy. No grade 3 or 4 non-hematologic toxicities, including sensory neuropathy, were observed. Moreover, no cardiac toxicity or treatment-related death occurred. Thus, 36.4% of patients experienced grade 3 or 4 toxicities, which included neutropenia-related adverse events. Four serious adverse events, including abdominal pain and neutropenia in 2 patients and febrile neutropenia in 2 patients, were reported. One patient was positive for HBsAg with HBV-DNA level of 2,849 IU/mL. The patient was given telbivudine as primary prophylaxis during and after R-CHOP chemotherapy and there was no evidence of HBV reactivation until last follow-up (Supplementary Table 2).

## DISCUSSION

Our current findings suggest that 6 cycles of chemotherapy may not be the gold standard for every DLBCL. Unlike cases of LS DLBCL treated with combined modality, in which chemotherapy is the main therapy and radiotherapy works as a consolidative treatment, the patients in this trial were treated with surgical resection followed by chemotherapy alone. In this surgico-chemotherapeutic approach, surgical resection removes the gross mass. Accordingly, the subsequent chemotherapy only needs to eradicate the microscopic disease whichever remains. Resected stage I Burkitt lymphoma can be managed with a short course of chemotherapy after surgical resection, even though it is the most aggressive form of B-cell non-Hodgkin's lymphoma [5, 6]. Hence, we supposed that 3 cycles of R-CHOP chemotherapy, without radiotherapy, might serve as an effective therapeutic approach for completely resected LS DLBCL.

Our previous retrospective study that included 18 completely resected DLBCL patients treated with a short course of chemotherapy also showed that none of the patients experienced disease progression or relapse [7]. The Memorial Sloan-Kettering Cancer Center group recently reported that LS DLBCL can be successfully managed irrespective of whether the therapeutic approach involves short or long-course chemotherapy, with or without radiotherapy [8]. In that study, 16 of 261 patients were treated with 3–4 cycles of R-CHOP chemotherapy without radiotherapy. Of these patients, 6 had fully resected disease. Although the specific outcomes in those resected patients were not illustrated separately, they achieved excellent outcomes; a 5-year PFS rate of 93.8% and 5-year OS rate of 100%.

We believe that a higher dose or a greater number of cycles of chemotherapy does not always translate into better outcomes. Eight cycles of CHOP or R-CHOP chemotherapy were previously used for the treatment of aggressive lymphoma; the number of cycles was reduced to 6 based on the findings of RICOVER-60, which compared 6 and 8 cycles of biweekly CHOP with or without rituximab [9]. Moreover, the risk-stratified therapeutic approach does not simply mean more intensive therapy for high-risk patients, but instead recommends a reduced intensity of therapy in the lower risk group to prevent attendant toxicities from overtreatment. The British Columbia Cancer Agency PET-based treatment algorithm recommends 4 cycles of chemotherapy in certain patients who achieve complete response after 3 cycles [10]. A similar risk-adapted therapeutic approach for LS DLBCL is also currently under investigation in the SWOG 1001 trial (Clinicaltrials.gov identifier NCT01359592). The DSHNHL-FLYER trial (NCT00278421) is an ongoing study assessing the non-inferiority of 4 cycles of R-CHOP, compared to 6 cycles, in low-risk LS DLBCL patients with an age-adjusted IPI of 0. The data on the exact threshold dose of chemotherapy for controlling DLBCL are insufficient. Surprisingly, we found that 2 elderly patients in our trial received a cyclophosphamide dose of only 1,312.5 mg/m^2^ and a doxorubicin dose of only 87.5 mg/m^2^, which corresponds to 1.75 cycles of full-dose R-CHOP as they underwent a 25% dose reduction from the first cycle, and the dose was further reduced to a half dose from the second cycle. These patients were disease-free and alive at the final follow-up.

Only 1 patient with primary splenic DLBCL experienced disease progression. He was the only patient with an IPI score of 2 and elevated LDH levels; in fact, his stage-modified IPI score was also 2, which corresponds to the intermediate risk group based on the LS DLBCL classification [3]. Even though the SWOG 8736 trial was conducted before the introduction of rituximab, the 5-year OS rates in patients with a stage-modified IPI of 2 was 60%, compared to the rates of 77% in those with 0 or 1 risk factor. The LDH level is a powerful prognostic marker that has been considered a surrogate of disease burden [11]. Furthermore, the patient exhibited not a solitary lesion but 2 nodules in the spleen. According to Han's criteria, 12 patients (54.5%) developed non-GCB type DLBCL, which is known to have a poor prognosis. However, none of these patients, except for the primary splenic case, experienced lymphoma relapse, which is consistent with previous report on the insignificance of COO in LS DLBCL treated with combined modality [8].

Surgical resection for aggressive lymphoma is primarily used for diagnosis and for treatment in cases of gastrointestinal lymphoma with pending perforation or intractable bleeding. Thus, we did not intentionally employ surgical resection for the treatment of localized lymphoma, but only selected those patients for abbreviated chemotherapy in this trial. However, some studies advocate that surgical resection combined with chemotherapy can help achieve better outcomes in patients with intestinal DLBCL [12, 13]. In particular, patients with T1 to T3 or N0 disease had significantly better OS, as compared to those with T4 or N1 disease [14]. However, regardless of LN metastases, depth of invasion (T1-3 vs. T4), or COO, all the patients with intestinal DLBCL survived without relapse until the last follow-up in the present study (Supplenetary Materials). This excellent outcome might be related to the patient selection, wherein only low-risk gastointestinal lymphoma patients with IPI of 0 or 1 have been selected. This finding suggests that certain low risk gastrointestinal lymphoma patients may achieve excellent survival without full-course R-CHOP chemotherapy.

Although R-CHOP is known to be a relatively tolerable regimen, it is still associated with grade 3 or 4 neutropenia in up to 80% of patients and febrile neutropenia in 20% of patients, as reported by recent phase 2 or 3 clinical trials (Table 2) [1, 15–17]. However, the incidence of these adverse event rates was lower in the present study, even without routine prophylactic G-CSF administration. In fact, grade 3 or 4 neutropenia was noted in 8 patients (36.4%) and grade 3 febrile neutropenia was noted in 3 (13.6%) patients; which can be understandably expected with less cumulative dose intensity although hematologic toxicities tend to occur more in the early period of treatment.

**Table 2:** Adverse events

Toxicity	G1	G2	G3	G4	All grades	G ≥ 3	AE, G ≥ 3 in the R-CHOP arm from other trials			
							LNH03-6B (n = 295) [16]	CRUKE/03/019 (n = 534) [17]	LYM-2034 (n = 79) [15]	SWOG 0014 (n = 60) [1]
All toxicity						8 (36.4%)	NA	380 (71%)	70 (89%)	54 (90%)
Hematologic										
Leukopenia	0	5	1	4	10 (45.5%)	5 (22.7%)	NA	NA	18 (23%)	NA
Neutropenia	0	3	1	7	11 (50.0%)	8 (36.4%)	189 (64%)	318 (60%)	64 (81%)	39 (65%)
Anemia	4	5	0	0	9 (40.9%)	0	51 (17%)	NA	7 (9%)	1 (2%)
Thrombocytopenia	1	1	0	0	2 (9.1%)	0	58 (20%)	28 (5%)	2 (3%)	2 (3%)
Febrile neutropenia	0	0	3	0	3 (13.6%)	3 (13.6%)	54 (18%)	58 (11%)	16 (20%)	9 (15%)
Non-hematologic										
Infection										
(no neutropenia)	3	0	0	0	3 (13.6%)	0	13 (4%)	125 (23%)	4 (5%)*	16 (27%)
Nausea	1	0	0	0	1 (4.5%)	0	3 (1%)†	20 (4%)	NA	17%
(all GI AE)										
Vomiting	0	0	0	0	0	0		17 (3%)	1 (1%)	
Diarrhea	0	1	0	0	1 (4.5%)	0	6 (2%)	NA	1 (1%)	
Anorexia	6	1	0	0	7 (31.8%)	0	NA	NA	NA	
Abdominal pain	2	2	0	0	4 (18.2%)	0	NA	NA	NA	
Constipation	2	0	0	0	2 (9.1%)	0	NA	NA	NA	
Mucositis	0	0	0	0	0	0	10 (3%)	10 (2%)	NA	NA
Fatigue	2	1	0	0	3 (13.6%)	0	NA	NA	NA	NA
Cardiovascular	0	0	0	0	0	0	25 (8%)	2 (< 1%)	1%‡ (n = 1)	5 (8%)
Sensory neuropathy	2	0	0	0	2 (9.1%)	0	16 (5%)	38 (7%)	2 (3%)	1 (2%)
Allergic reaction	1	0	0	0	1 (4.5%)	0	0 (0%)	NA	NA	NA
Skin rash	1	1	0	0	2 (9.1%)	0	1 (< 1%)	NA	NA	1 (2%)
**Treatment-related mortality**						0	14 (5%)	3 (1%)	3 (4%)	1 (2%)

Abbreviation: AE, adverse events; G, grade; GI, gastrointestinal; NA, not available

SWOG 0014 was a single arm study of 3 cycles of R-CHOP and radiotherapy.

*pneumonia

^†^Nausea or vomiting

^‡^One on-study cardiac arrest

Considering the favorable prognosis of LS DLBCL, long-term morbidity and non-relapse mortality should also be considered in the therapeutic decision. In the SWOG 8736 trial, 17.9% of patients reported a second malignancy after treatment and approximately 10% died as a result of this malignancy [18]. Moreover, 14% of patients in the GELA LNH-93 study died due to a second malignancy. However, no cases of death due to second malignancy were noted in our study, as expected, given the low cumulative chemotherapy dose and the short follow-up period. Nevertheless, long-term follow-up is necessary. In addition, cardiovascular disease is another long-term toxicity to consider. In the SWOG 8736 trial, the incidence of cardiovascular events was 16.5%, with a higher rate of mortality in the chemotherapy alone arm (21.4% vs. 11.6%) [18]. The development of anthracycline-related cardiotoxicity is well-known to be strongly correlated with the cumulative dose [19]. Even though cardiotoxicity after R-CHOP chemotherapy is not as common, as the cumulative dose of doxorubicin is limited to 250 mg/m^2^ (well below the usual recommended upper limit dose of 450–500 mg/ m^2^), 1–4% of patients were found to experience grade 3 cardiotoxicity, even in recent trials, as there is variable sensitivity to anthracyclines among patients [15–17, 20]. The mean cumulative dose of doxorubicin was 139.3 ± 20.2 mg in the present study, which corresponds to 46.4% of the full dose in the 6 cycles of R-CHOP chemotherapy.

The present study is a small-scale phase 2 trial, and hence, the findings need to be cautiously interpreted. In particular, this included a very small number of nodal DLBCL cases. Nevertheless, it is well-known that stage 1 nodal DLBCL has better prognosis than extranodal disease, including intestinal lymphoma, which has the best prognosis among primary extranodal lymphomas (median OS of nodal DLBCL vs. intestinal DLBCL: 180 vs. 159 months) [21]. Nevertheless, our findings should be confirmed in a larger study even though the efficacy outcome was excellent. Moreover, the median follow-up duration was 39.5 months. This is not a short length of follow-up, considering that patients achieving 2-year EFS are known to have an OS equivalent to that of the age- and sex-matched general population [22]. However, the recently published long-term follow-up data of SWOG 8736 showed that the continued risk of relapse exists in LS DLBCL, unlike that with advanced stage disease [18]. Accordingly, a longer follow-up duration would more definitely indicate the actual prognosis in these patients.

To our knowledge, this is the first prospective clinical trial to suggest a reduced number of immunochemotherapy cycles is feasible for completely resected DLBCL. However, these findings should not be generalized to all cases, including those with disease at high-risk sites such as the testis, breast, orbit/nasal/sinus region, or central nervous system, and patients with bulky disease. Despite these limitations, we conclude that 3 cycles of abbreviated R-CHOP immunochemotherapy might be effective and safe for patients with localized and completely resected DLBCL, particularly among those with low-risk IPI, which warrants further validation.

## MATERIALS AND METHODS

### Study design and participants

This prospective, single-arm, phase 2 trial was conducted in 13 hospitals in Korea. Eligible patients were aged ≥ 18 years, had newly diagnosed stage 1 or 2 CD20-positive DLBCL, and had undergone complete resection of all lesions. Completely resected disease was defined as the lack of any residual lymphoma on spiral CT of the involved area after surgical resection, or confirmed complete excision with negative resection margins on pathologic examination after surgery in the case of stage I disease with a single lesion on preoperative imaging study. All the pathological results were reviewed by a dedicated pathologist (J Huh), and the lesions were confirmed to be de novo DLBCL as per the WHO classification [23]. Other eligibility criteria included Eastern Cooperative Oncology Group (ECOG) performance status of 0–2 and adequate hematologic and chemistry profiles. The exclusion criteria included bulky disease; primary breast, testicular, or central nervous system lymphomas; any previous lymphoma treatment except for surgical resection; HIV seropositivity; B symptoms; or any disorder that would compromise the patient or the study, in the opinion of the investigator. The study was reviewed and approved by the institutional review board at each participating institute and was registered at ClinicalTrials.gov (NCT01279902).

### Procedures

Baseline assessments included complete blood count, determination of the serum lactate dehydrogenase (LDH) levels, bone marrow aspiration and trephine biopsy, CT scanning of the involved lesions and FDG-PET. All these studies were performed before R-CHOP chemotherapy and after the completion of 3 cycles of immunochemotherapy; to monitor relapse, these examinations, except for FDG-PET, were repeated at 3-month-intervals for 2 years, and at 6-month-intervals for the next 3 years. R-CHOP chemotherapy was initiated within 6 weeks from the surgical resection and was repeated every 3 weeks over 3 cycles [24]. The upfront dose reduction of doxorubicin and cyclophosphamide by 25% was permitted if the patients were aged > 65 years, at the discretion of investigators. Prophylactic G-CSF was not routinely administered.

### Outcomes

The primary endpoint was 2-year disease-free survival (DFS), defined as the time from the date of surgical resection to the first recording of relapse or death by any cause. Secondary endpoints included overall survival (OS), defined as the time from surgical resection to death or the last follow-up, and safety. The treatment response was assessed according to the International Working Group response criteria [25]. Toxicity was evaluated before each treatment cycle according to the National Cancer Institute Common Toxicity Criteria (NCI CTC) version 3.0.

### Statistics

This study required the enrollment of 20 assessable subjects in order to determine whether the 2-year DFS rate is ≤ 70% (P0) or ≥ 90% (P1), with an alpha level of 0.05 and a power of 80% using a two-sided test. We assumed that the drop-out rate would be 10%, and hence, a total of 23 patients were required. All patients who received any treatment in the study, except for those who were ineligible, were included in the treatment evaluation, safety, and efficacy analyses. The Kaplan–Meier method was used to calculate the DFS and OS. All statistical analyses were performed using SPSS version 23.

## SUPPLEMENTARY FIGURE AND TABLES


