# Supplementary material for: Feasibility of abbreviated cycles of immunochemotherapy for completely resected limited-stage CD20+ diffuse large B-cell lymphoma (CISL 12-09)
Source: Oncotarget. 2017 Jan 5;8(8):13367–74. doi: 10.18632/oncotarget.14531 (PMC5355104; doi:10.18632/oncotarget.14531)
Supplement: Supplementary file 1 [file oncotarget-08-13367-s001.pdf]

Feasibility of abbreviated cycles of immunochemotherapy for completely resected limited-stage CD20+ diffuse large B-cell lymphoma (CISL 12-09)

Supplementary Materials

Subgroup analysis of GI tract disease

Among the 16 patients with GI tract involvement, the primary sites included the stomach (*n* = 1), jejunum (*n* = 1), ileum (*n* = 10), ileocecal valve (*n* = 3), and appendix (*n* = 1) (Supplementary Table 1). The median age at diagnosis was 54 years (range, 31–77 years). Eight patients had stage 2 disease according to Ann-Arbor staging, whereas 9 were classified as stage 2 according to Lugano staging; 1 patient without LN involvement showed invasion to the mesenteric fat, which corresponds to II-E according to Lugano staging and T4 according to Paris staging. Seven patients had T3 or T4 disease according to Paris staging. One patient had terminal ileum and mesenteric LN involvement, which was classified as stage II-2 according to Lugano stating and T3N1 according to Paris staging. The preoperative LDH level was available in 9 patients, and was found to be elevated in 2 patients. The preoperative IPI scores were 0 in 7 and 1 in 2 patients. The stage-modified IPI was 2 in only 1 patient. When classified according to the COO, 6 patients were classified as having GCB-DLBCL and the remaining 10 were classified as having non-GCB DLBCL. As all the patients survived without disease progression, the estimated 2-year DFS and OS rates were 100.0% in this subgroup (Supplementary Figure 1).

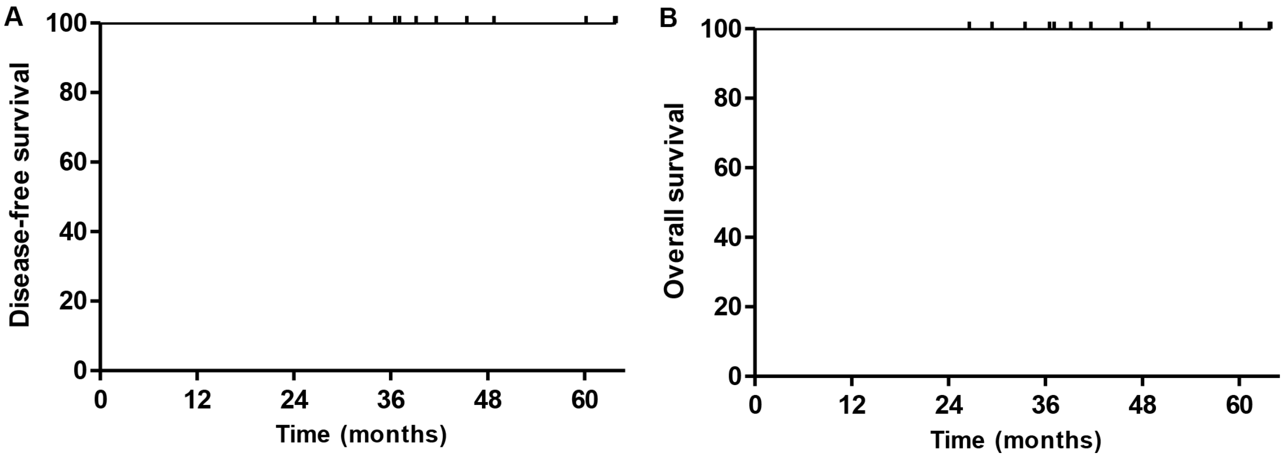

Supplementary Figure 1: Kaplan-Meier curves of (**A**) disease-free survival (DFS) and (**B**) overall survival (OS) of patients with gastrointestinal tract involvement. The estimated 2-year DFS and OS rates were 100.0% in this subgroup.

Supplementary Table 1: Characteristics of patients with gastrointestinal tract involvement

Clinical Characteristics	*n* = 16
Time to chemotherapy after resection, days (median, range)	28 (12–49)
Age, years (median, range)	54 (31–77)
≤60	10 (62.5%)
>60	6 (37.5%)
Sex	
Male	14 (87.5%)
Female	2 (12.5%)
ECOG performance status,	
0–1	16 (100.0%)
Ann Arbor stage	
1/1E	8 (50.0%)
2/2E	8 (50.0%)
Paris staging	
T2N0	4 (25.0%)
T2N1	3 (18.8%)
T3N0	3 (18.8%)
T3N1*	4 (25.0%)
T4N0	1 (6.3%)
TxN1	1 (6.3%)
Lugano staging	
I	7 (43.8%)
II–1	7 (43.8%)
II–2*	1 (6.3%)
II–E	1 (6.3%)
Lactate dehydrogenase, preoperative	
Within normal limit	8 (50.0%)
Increased	1 (6.3%)
NA	7 (43.8%)
IPI, preoperative	
0	7 (43.8%)
1	2 (12.5%)
NA	7 (43.8%)
Stage-modified-IPI, preoperative	
0	2 (12.5%)
1	6 (37.5%)
2	1 (6.3)
NA	7 (43.8%)
Involved sites	
Small and/or large intestine	15 (68.2%)
Without lymph node involvement	7 (46.7%)*
With lymph node involvement	8 (53.3%)*
Stomach	1 (4.5%)
Cell of origin according to Han's criteria	
GCB	6 (37.5%)
Non-GCB	10 (62.5%)

*One patient had mesenteric lymph node involvement.

Abbreviation: ECOG, Eastern Cooperative Oncology Group; GCB, germinal-center B-cell like; IPI, International prognostic index; NA, not available .

Supplementary Table 2: HBV-related markers

**Markers**	***n* = 22**
HBsAg	
Positive	1* (4.5%)
Negative	21 (95.5%)
HBsAb	
Positive	17 (77.3%)
Negative	5 (22.7%)
HBcAb	
Positive	8 (36.4%)
Negative	8 (36.4%)
Not done	6 (27.3%)
HBV-DNA	
Positive	1* (4.5%)
Negative	3 (13.6%)
Not done	18 (81.8%)

* One patient was positive for HBsAg with HBV-DNA level of 2,849 IU/mL. The patient was given telbivudine as primary prophylaxis during and after R-CHOP chemotherapy and there was no evidence of reactivation until last follow-up.
